# Microencapsulation of *Lactobacillus acidophilus* LA‐5 and *Bifidobacterium animalis* BB‐12 in pectin and sodium alginate: A comparative study on viability, stability, and structure

**DOI:** 10.1002/fsn3.2470

**Published:** 2021-07-20

**Authors:** Zahra Motalebi Moghanjougi, Mahmoud Rezazadeh Bari, Mohammad Alizadeh Khaledabad, Saber Amiri, Hadi Almasi

**Affiliations:** ^1^ Department of Food Science and Technology Factually of Agriculture Urmia University Urmia Iran

**Keywords:** freeze‐drying, gastrointestinal, hydrogels, microencapsulation, probiotics

## Abstract

The present study aimed at examining whether the microencapsulation of *Lactobacillus acidophilus* LA‐5 and *Bifidobacterium animalis* BB‐12 inside hydrogels could prolong their survival in freeze‐drying conditions, stored at 4℃ and in the gastrointestinal medium. Microencapsulation was performed by emulsion with a syringe, while sodium alginate and high methoxyl pectin were used as a carrier material. A relatively high efficiency of encapsulation was obtained (>92%). Z‐Average and pdI in samples were not significant (*p* < .05). In different treatments, changes in the number of bacteria after freeze‐drying, 30 days of storage, and gastrointestinal conditions, compared to each other, were significant (*p* < .05). However, the survival rate after a reduction during storage was higher than 10^6^ cfu/g, indicating the suitability of the microencapsulation process. The surface of microcapsules observed by a scanning electron microscope (*SEM*) confirmed the success of encapsulation. Finally, a lower decrease in the count of microencapsulated was observed in comparison to the free cells.

## Graphical abstract

Microencapsulated bacteria have many advantages over free cells, including protection of biocatalysts, high volume of productivity, improved control process, protection of cells against damages and reduced sensitivity to contamination. However, stabilization of probiotic cells requires some specific processes that have complex stages of food production and increase its cost. Results of this study about the effectiveness of encapsulation to protect probiotics are controversial, and the high diversity of parameters under evaluation makes it difficult to find the best method of encapsulation.

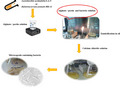

## 1 INTRODUCTION

The most common probiotics introduced into functional foods are *Lactobacillus* and *Bifidobacteria* species, known as a nonpathogenic resident of the intestine, playing an important role in preventing the colonization of pathogens and the adjustment of host safety response (Amiri et al., 2020; de Lara Pedroso et al., 2012; Sohrabpour et al., 2021). *Bifidobacterium lactis* BB‐12 and *Lactobacillus acidophilus* LA‐5 are two commercial probiotic strains widely used as adjuvant cultures and generally known as "safe" (GRAS) (Amiri et al., 2020, 2021). *L. acidophilus* shows antimicrobial effect due to the formation of organic acids and bacteriocin. It is also resistant to bile acid and has an antibiotic effect on intestinal pathogens such as *Escherichia coli, L. acidophilus* can attach to the intestine and survive for 2 days in the gastrointestinal juice. *Bifidobacteria* are used because they produce low acid and consume more lactic acid during storage. They have probiotic properties such as anti‐cancer activity, folic acid synthesis, improvement of the nutritional value of food, and induction of immunoglobulin production. *B*. *animalis* is mostly selected for fermented dairy products because of its beneficial effects on human health and oxygen and acid tolerance compared with other species. *B*. *animalis* BB12 is capable of simultaneously producing conjugated linoleic acid, exopolysaccharides, and bacteriocins as postbiotics (Amiri, Aghamirzaei, et al., 2021; Amiri et al., 2021). Probiotics must be resistant to food operating, storage, and intestinal conditions to reach their intended location and show health effects with a minimum amount 10^6^–10^7^ cfu/g (Amiri et al., 2021; Mularczyk et al., 2021; Rezazadeh‐Bari et al., 2019; Vallejo‐Castillo et al., 2020).

Microencapsulation is an acceptable method for probiotics protection, which provides high survival and high performance due to controlled release. Extrusion, spray‐drying, and emulsion are the most common methods for probiotic microencapsulation; extrusion and spray‐drying are less used, owing to probiotic susceptibility to applied temperatures and large particle size (Amiri et al., 2021; Liu et al., 2017; Martin et al., 2013; Nasri et al., 2020; Ohlmaier‐Delgadillo et al., 2021; Saini et al., 2020; dos Santos et al., 2019).

Microencapsulation by emulsification/internal ionic gelation is a suitable method for the production of water in oil emulsion particles, described for the stabilization of unstable materials (Holkem et al., 2017). One advantage of this method is that smaller particles (less than 100 μm) do not alter the sensory properties of the product. This method requires no special equipment and sophisticated techniques, and due to its simple formulation and low cost, it has high cell viability and porous particles (Amine et al., 2014; Gebara et al., 2013; Holkem et al., 2016).

Alginate is the major compound used for the microencapsulation of probiotics, mainly because of its safety, good gelling properties (temperature and pH), and biocompatibility. Alginate is degraded in low pH, allowing the release of probiotics in digestive conditions (Amine et al., 2014; Han et al., 2018; Martin et al., 2013; Pupa et al., 2021; Qi et al., 2020; Sánchez‐Portilla et al., 2020).

Pectin is a nontoxic and cheap polymer that forms a gel structure in the presence of divalent metal ions such as calcium. In the encapsulation process, the use of high methoxyl pectin is more efficient; high molecular weight and high gelling power provide small microparticles (Awasthi, 2011; Fathi et al., 2014; Panghal et al., 2019).

Particle size is an important factor, since large grains may produce sandy texture in the product, while small grains do not provide sufficient protection for bacteria. Therefore, probiotics should be trapped in a limited range of particle sizes to minimize the problems associated with cell survival and food texture (Machado et al., 2020). Resistance to gastrointestinal conditions depends on the strain and species. The selection of carrier matrix can improve survival and significantly increase the number of live bacteria reaching the colon (Yonekura et al., 2014).

Therefore, the objective of this study was to produce probiotic microcapsules of *L. acidophilus* LA‐5 and *B*. *animalis* BB‐12 with emulsion technique in the sodium alginate and pectin with freeze‐drying and the evaluation of cell survival after the process and stability under gastrointestinal simulation conditions and their viability during 30 days of storage at a refrigerated temperature.

## 2 MATERIALS AND METHODS

### 2.1 Materials

The lyophilized culture of *B*. *animalis* subsp *lactis* BB‐12 and *L. acidophilus* LA‐5 (Christian Hansen, Hoersholm, Denmark), sodium alginate (Sigma‐Aldrich, Saint Louis, Missouri, USA), high methoxyl citric pectin (Sigma‐Aldrich GmbH, Sternheim, Germany), Canola oil (Famila, Tehran, Iran) were purchased. Other utilized products included Tween 80, Calcium Chloride Dihydrate, Lithium chloride, L‐cysteine, Sodium citrate, Sodium Chloride, Peptone water, Hydrochloric Acid, Monopotassium phosphate (Sigma‐Aldrich, Saint Louis, Missouri, USA), MRS agar, MRS broth (Merck, Darmstadt, Germany), and gas pack (Anaerocult A, Darmstadt, Merck).

### 2.2 Methods

#### 2.2.1 Preparation of probiotic bacteria

Lyophilized culture of *L. acidophilus* LA‐5 and *B*. *animalis* BB‐12 was inoculated into 10 ml MRS broth and MRS broth containing 0.05%, L‐cysteine Hydrochloride, and 0.1% Lithium Chloride (MMRS broth), respectively, and incubated for 48 hr at 37℃. *Bifidobacterium* was incubated under anaerobic conditions by a gas pack system. The cultivation was repeated to reach the required number of bacteria. Then, probiotic cells were separated by centrifuge (D78532, Hettich, Germany) at 1792*g* for 15 min at 4℃. The bacteria were washed twice with sterile physiology serum (Moghanjougi et al., 2020).

#### 2.2.2 Microencapsulation procedure

##### Microencapsulation in sodium alginate

Microencapsulation of bacteria in sodium alginate was carried out according to the emulsion method developed by Holkem et al. (2016) with some modifications. First, sodium alginate solution (2% w/v) was prepared in deionized water and after sterilization with an autoclave (C73981, Webwco, Germany) stored in a refrigerator for 24 hr, so that alginate particles were well absorbed. The next day, to coincide with the environment temperature, alginate solution was transferred to the outside of the refrigerator. Then, in a sterile condition, 5 ml of microbial suspension was mixed with 20 ml of sodium alginate, then added by sterile syringe as a dropper into a solution containing 99 g of rapeseed oil and 1 g of Tween 80 (previously sterilized), blended using a magnetic stirrer (RS3001, MLW, Germany) at 750 rpm and placed in the same round for 20 min until the mixture was completely emulsified in the oil phase. After that, 40 ml of sterilized calcium chloride solution (0.1 M) was added to the emulsion solution by syringe as a dropper and then emulsion was mixed on a magnetic stirrer for 5 min at 100 rpm. Due to the contact of alginate with calcium solution, the capsule wall was formed and beads were sedimented at bottom of the container. After completion of mixing time, 40 ml of sterile peptone water was added to separate the phases, and the solution was stabled for 30 min. After the complete sedimentation, the oily layer was poured out and microcapsules were separated by centrifugation at 324*g*, and temperature of 4℃ for 10 min. The beads were rinsed twice with sterile physiology serum (0.9%) to remove residual particles. In the end, microcapsules were kept in sterile‐sealed containers with peptone water at refrigerator temperature until later use.

##### Microencapsulation in pectin

Microencapsulation in pectin was performed using the emulsion method provided by Gebara et al. (2013) with some modifications. About 2 g of pectin powder was added to 100 ml of distilled water twice at 70℃ and was stirred continuously with a magnetic stirrer until it was completely dissolved. The solution was sterilized by filtration set (Millipore, Merck, Germany) with a filter paper size of 0.88 μm. Other steps were similar to the alginate method, with a difference that 0.8 M solution of calcium chloride was used.

### 2.3 Probiotic cell count

Microcapsule cell counting was fulfilled by the method provided by Holkem et al. (2016) with some modifications. One ml of microcapsules was added to 9 ml of sterile sodium citrate solution (2% w/v, pH 7), and it was homogenized by a stomacher (Circulator400, Seward, UK) at 260 rpm for 4 min. During this process, beads were destroyed and bacterial cells were released. Serial dilution step with sterile peptone water solution (0.1%) was performed using pour plate method in MRS Agar medium. Finally, the number of bacteria was counted after 37 hr of incubation at 37℃. For free cell count, the pour plate technique was performed according to the method provided by de Lara Pedroso et al. (2012) with some modifications. It should be noted that *Bifidobacterium* was inoculated in the MRS Agar medium and incubated in anaerobic jars using the anaerobic gas pack system. All plates were done in two repetitions.

### 2.4 Encapsulation efficiency

The efficiency of encapsulation, showing the number of living microorganisms during the microencapsulation process, was calculated using Equation 1 (Maleki et al., 2020):

(1) $$\%EE=\left(\left.\frac{N}{N_{0}}\right)\times 100\right.$$

Where EE% is the percentage of the efficacy of capsulation; N denotes the number of cells released from capsules (cfu/g) and N_0_ represents the number of live cells used for encapsulation (cfu/g).

### 2.5 Evaluation of the stability of microcapsules to Freeze‐Drying

To evaluate of freeze‐drying effect, on the same day, a portion of microcapsules was frozen at −18℃ for 24 hr. The frozen microcapsules were dried in a vacuum dryer (FD‐5005‐BT, Dena industry, Iran).

### 2.6 Stability of microencapsulated bacteria during storage

The microencapsulated bacteria were stored in sterile peptone water in a 1:1 ratio at 4℃ for 30 days, and the survival rate was assessed using the method outlined in the previous sections (Martin et al., 2013).

### 2.7 Survival of probiotics after exposure in gastrointestinal conditions

The test was carried out using the method developed by Maleki et al. (2020) with a few changes. One gram of freshly prepared beads was added to 10 ml simulated gastric juice (GJ) (HCl 0.08 M containing 0.2% NaCl, pH 1.55), without pepsin and incubated at 37℃ for 0, 60, and 120 min. After incubation, 1 ml of the above solution was removed and placed in 9 ml of simulated intestinal juice (IJ) without bile salts (KH_2_PO_4_, pH 7.43) and incubated at 37℃, for 50, 100, and 150 min. After incubation, 1 ml of the solutions was pure plated using the method described in the probiotic cell count section.

### 2.8 Characteristic of microcapsules

#### 2.8.1 Particle size analysis

Dynamic light scattering (DLS) is a physical method used to determine the distribution and other particles in solutions and suspensions based on their Brownian motion. First, 2 ml of samples was poured into a cuvette and diluted with distilled water twice for distillation. Then, the cuvette was placed in a dynamic diffraction analyzer (Nano ZS ZEN 3,600, Malvern, UK), and parameters were measured using visible light with a wavelength of 633 nm at 25℃.

#### 2.8.2 Morphological characteristics

For observation of the morphology of microcapsules, the surface of microcapsules with different magnifications was characterized by an *SEM* (LEO1430VP) at room temperature. The electron was reflected to the surface of the sample coated with gold in the vacuum environment, then collected by the detector and transformed into an optical photon to create a visible image.

#### 2.8.3 Experimental design and data analysis

All experiments were carried out in a completely randomized design with three replications. Analysis of variance was done at α = 0.05, and the least significant difference test was used to confirm the difference between the means at *p* < .05 using Microsoft Excel 2016 software.

## 3 RESULT AND DISCUSSION

### 3.1 Encapsulation efficiency

**FIGURE 1:**
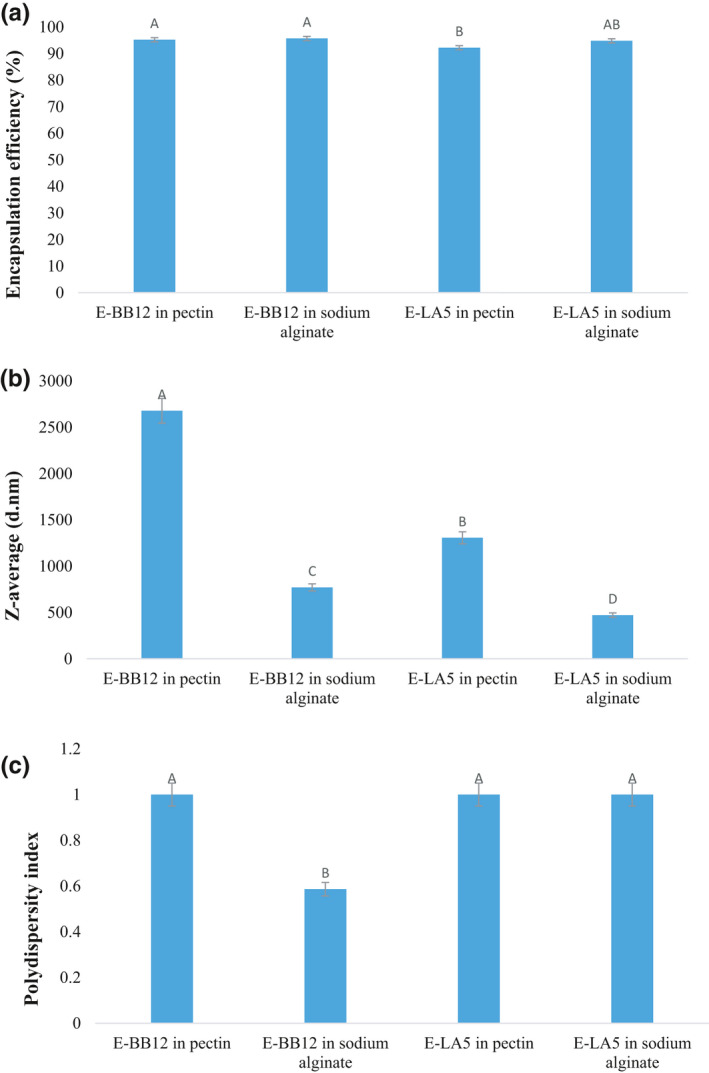
Encapsulation efficiency (a), Z‐average (b) and pdI (c) (E‐BB12: Encapsulated *B*. *animalis* BB‐12, E‐LA5: Encapsulated *L. acidophilus* LA‐5)

The results of variance analysis showed that the efficiency of encapsulation was not significant in different samples (*p* > .05) (Figure 1(a)). The encapsulation efficiency obtained in this study was similar to (Gebara et al., 2013; Holkem et al., 2016; Krasaekoopt et al., 2004) results. They reported, respectively, the average efficiency of 89, 84, and 99% for pectin microcapsules and sodium alginate containing *L*. *acidophilus* and *B*. *animalis* by the internal gelatinization method. It was observed that the size of pectin microcapsules was higher than that of sodium alginate, probably related to wall material and the high viscosity of 2% (W) of pectin solution relative to the same amount of sodium alginate (Colín‐Cruz et al., 2019; Sandoval‐Castilla et al., 2010; Yonekura et al., 2014), corresponding to Sandoval‐Castilla et al. (2010) results. It can be said that encapsulation efficiency can be influenced by various factors such as the type of wall and its concentration and the concentration of calcium chloride used in the formulation, the species of the encapsulated microorganisms, the method used, and the particle size (Hugues‐Ayala et al., 2020). In general, the obtained results indicated that bacterial damage during the microencapsulation process by the emulsion method was low; therefore, it seemed to be a practical and appropriate method.

### 3.2 Evaluation of the stability of microcapsules to Freeze‐Drying

**FIGURE 2:**
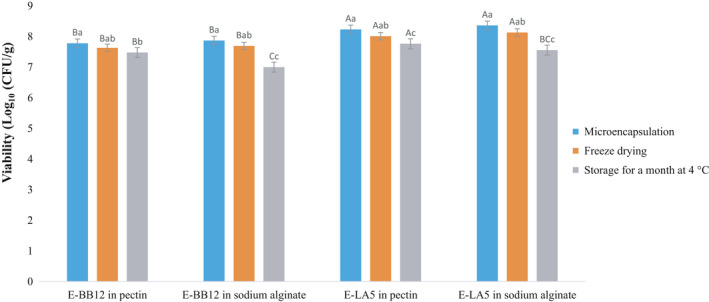
Viability of bacteria to microencapsulation, freeze‐drying and storage for a month at 4℃, (E‐BB12: Encapsulated *B*. *animalis* BB‐12, E‐ LA5: Encapsulated *L. acidophilus* LA‐5)

In different treatments, changes in the number of bacteria after freeze‐drying and compared with each other were significant (*p* < .05) (Figure 2). *B*. *animalis* BB‐12 was more susceptible to freeze‐drying than *L. acidophilus* LA‐5, and pectin microcapsules exhibited higher resistance to freeze‐drying conditions; however, reduction of alive cells in microcapsules by emulsion method was low. There are many documents on the beneficial effects of microencapsulation on bacterial survival after freezing. Amine et al. (2014) reported that in the small capsules, the viability of bacteria increased during freeze‐drying in peptone medium. The survival level after freeze‐drying was different due to several factors including the bacterial strain, the effect of protective compounds, the difference in the cell wall, and membrane compounds (Jagannath et al., 2010).

### 3.3 Stability of microencapsulated bacteria during storage

The number of live bacteria in different treatments was significantly changed after 30 days of storage (*p* < .05) (Figure 2). The stability of *B*. *animalis* BB‐12 microcapsules was better. These results were similar to those of de Lara Pedroso et al. (2012). The probiotics exposed to moisture, oxygen, and heat cause irreversible damage to the microbial cells. Products in liquid form often exhibit less sustainability than other forms of storage at low temperatures, indicating a tendency to reduce the viability of storage during the time. Probiotic bacterial strains kept at room temperature or in the refrigerator for 6 months showed a decrease in viability, but viability in refrigerated samples was more than that at room temperature; therefore, cellular stability increased with a decrease in temperature because, at low temperatures, the exposure of active compounds to the bacterial cell prevented and prolonged the useful life of microcapsules (Albertini et al., 2010; Holkem et al., 2016). Loss of survival during storage can be related to some factors such as the formation of free radicals in presence of oxygen, the oxidation of fatty acids, and DNA damage (Holkem et al., 2017; de Lara Pedroso et al., 2012). Although cellular damage and loss of viable count during storage and processing occur, a proper microencapsulation process should ensure the survival of bacteria in these stages, with a minimum live probiotic of about 10^6^ cfu/g (Holkem et al., 2017). However, in this study, the survival rate during storage time was higher than this value.

### 3.4 Probiotic bacterial survival in gastrointestinal conditions

**FIGURE 3:**
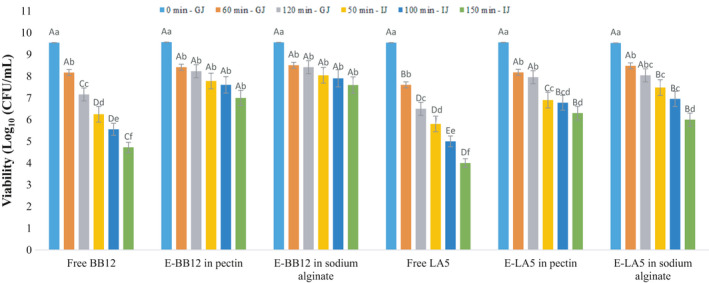
Probiotic bacterial survival in gastrointestinal conditions (E‐BB12: Encapsulated *B*. *animalis* BB‐12, E‐ LA5: Encapsulated *L. acidophilus* LA‐5)

The results of data analysis in gastrointestinal conditions showed no significant difference among different treatments (*p* < .05) (Figure 3). At different times of gastrointestinal conditions, the number of bacteria significantly decreased (*p* < .05). Results indicated that *L. acidophilus* LA‐5 and *B*. *animalis* BB‐12 had similar resistance in these stress conditions. With incubation in gastrointestinal conditions, a significant decrease in survival of free cells in comparison with the encapsulated cells was observed, consistent with the results reported by Gebara et al. (2013) studies. The viability of probiotics represents the high efficiency of the production method. A reduction in the size of microcapsules can probably increase gastrointestinal resistance (Albertini et al., 2010). The ultimate survival of probiotic bacteria in the colon is at least 10^7^ cfu/g, and microencapsulated alive probiotics can transfer from stomach to intestine. At the neutralized pH of the intestinal, the microcapsules can dissolve, leading to probiotics release, Therefore, the results showed that the microencapsulation of bacteria limited the inhibition of acid, leading to an increase in the viability of the microencapsulated cultures than free cells. The protective effect of alginate on the survival of probiotic bacteria has been confirmed in gastrointestinal stress conditions (Amine et al., 2014; Ramos et al., 2018).

In the present study, free and the microencapsulated cells of *B*. *animalis* BB‐12 were resistant to simulated gastric juice and simulated intestinal juice during 270 min; however, free cells of *L. acidophilus* LA‐5 decreased during 220 min, and they survived over 270 min in encapsulated forms. This difference could be related to strains differences in tension conditions. It can be said that microencapsulation of *L. acidophilus* LA‐5 can provide better protection. These results were consistent with those of de Lara Pedroso et al. (2012). The reason for the increase in the number of bacteria in the gastrointestinal tests is the disintegration of microparticles and the release of probiotics. In the case of *B*. *animalis* BB‐12 in sodium alginate microcapsules, in IJ medium the pH increased; therefore, the number of live cells would increase. It can be said that alginate, under acidic conditions, was converted to insoluble alginic acid, preventing the active compounds from penetrating, while the alkaline environment led to the dissolution of alginate and breaking of microparticles. Therefore, microencapsulation with alginate was suitable for the survival of probiotics under acidic conditions (Holkem et al., 2017). Besides, pectin nanoparticles were resistant to acidic and enzymatic conditions, so those microcapsules could enter the colon environment (Fathi et al., 2014).

The results of this study confirmed the effectiveness of microencapsulation in protecting probiotics against digestive conditions, but some factors such as microorganisms, microencapsulation conditions, and various methods for evaluating the effectiveness of encapsulation such as pH, presence or absence of enzymes, and various wall materials can provide different results.

### 3.5 Particle size analysis

The results of analysis of Z‐Average and pdI in different samples were insignificant (*p* > .05). Due to the use of a similar type of syringe in the production of microcapsules, the type of coating material did not affect particle size. In general, the microcapsules of pectin are larger than sodium alginate, and microcapsules *B*. *animalis* BB‐12 also has a larger particle size than *L. acidophilus* LA‐5 (Figure 1b,c). These results were consistent with *SEM* images, confirming the largeness of pectin microcapsules containing *B*. *animalis* BB‐12 compared with other particles. In the study by Sandoval‐Castilla et al. (2010), the size of alginate microcapsules was smaller than pectin.

Due to the use of a low diameter syringe, the average diameter of particles was small in comparison to results obtained in other studies for the microencapsulation by emulsion method. Barbosa et al. (2015) reported the mean diameter of alginate microcapsules containing *Lactobacillus corvatus* by emulsion method at about 266 and 473 μm. The diameter of *Bifidobacterium* in sodium alginate microcapsules by emulsion method performed by Hansen et al. (2002) and Holkem et al. (2017) was reported to be about 19, 67, and 54 μm, respectively. In addition, the diameter of *L. acidophilus* and *B*. *bifidum* microcapsules in sodium alginate 2% by Krasaekoopt et al. (2004) was reported to be 1.6 μm, roughly similar results.

The low mean diameter of particles can be attributed to the high efficiency and effect of the presence of probiotics in microparticles, and this effect can be ascribed to a change in the zeta potential of microcapsules, as reported by Martin et al. (2013). They reported that alginate microcapsules containing probiotics had a smaller size than nonprobiotic microcapsules. The application of the emulsion method to produce microcapsules could control the size of gelatinization and similar microparticles; the diameter of microparticles was controlled by the concentration and viscosity of sodium alginate and pectin solutions and the mixture of emulsion (Hansen et al., 2002). The size of microparticles affects the efficiency of encapsulation and food texture. The diameters smaller than 100 μm are preferred for most applications for better protection against the gastrointestinal tract (Holkem et al., 2017; Mirtič et al., 2018).

### 3.6 Morphological characteristics

**FIGURE 4:**
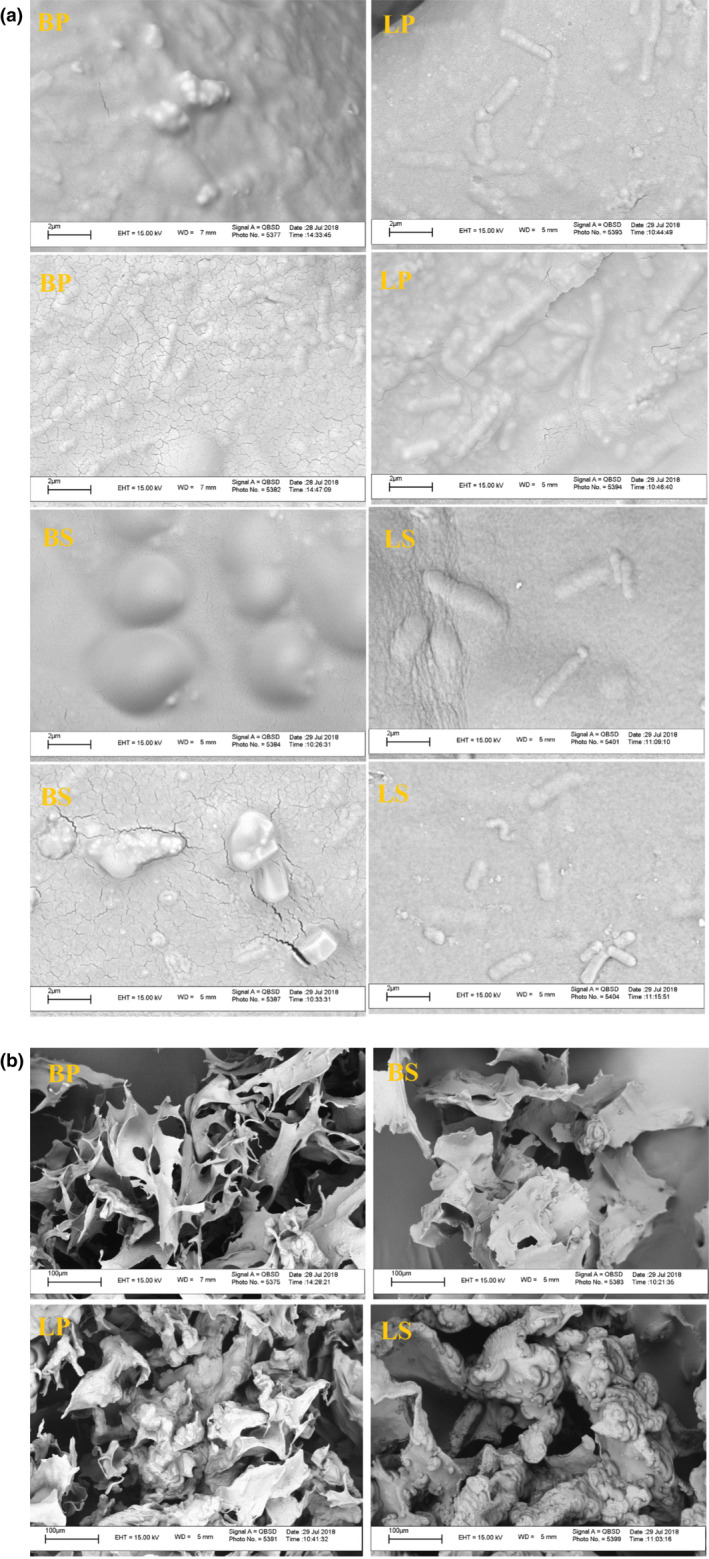
*SEM* images of microcapsules (BP: *B*. *animalis* BB‐12 in pectin, BS: *B*. *animalis* BB‐12 in sodium alginate, LP: *L*. *acidophilus* LA‐5 in pectin, LS: *L*. *acidophilus* LA‐5 in sodium alginate)

According to Figure 4a,b, elliptic microcapsules were similar to the results obtained by Jagannath et al. (2010). The rugged surface of microcapsules indicates the presence of probiotics inside the capsules (Martin et al., 2013). It was also observed that the immobilization of cells in sodium alginate produced semispherical microcapsules (shape BS) with rigid surface and spongy structure (Albertini et al., 2010; Hansen et al., 2002). Kinetics of gelatinization can create a capsular‐like structure in alginate particles, determined by concentration and rate of Ca^2+^ penetration, the structure and concentration of alginate, and the presence of Na^+^ ions preventing alginate gelatinization. The presence of Ca^2+^ and Na^+^ ions also promotes the formation of a homogeneous gel. Inside microcapsules is made up of a semiporous network. Alginate porosity is important in keeping bacteria alive when they pass through gastrointestinal tract (Allan‐Wojtas et al., 2008).

The cavities all over the microcapsules were due to the rapid submersion of frozen water from the microcapsule matrix during the freeze‐drying process, leading to porosity in places where there were ice crystals. Particles were accumulated together because of their fineness. These images were similar to results obtained by Holkem et al. (2016). Generally, wrinkles and cracks are the results of the mechanical stress caused by nonuniform drying of various parts of the liquid droplets in the early stages of drying. High molecular weight polymers dry quickly to prevent the release of internal vapors, resulting in increased bubble formation in the matrix of wall materials, expanding the internal space of the microcapsule, and creating more concavity (Maleki et al., 2020). Comparison of pectin and alginate microcapsules showed that alginate beads were relatively spherical, while pectin beads had a geometrically shaped plate; this phenomenon was related to the difference in the cross‐links created in each case.

## 4 CONCLUSION

Microencapsulated bacteria show many advantages over free cells, including protection, high volume of productivity, improved control process, protection of cells against damages, and reduced sensitivity to contamination. However, the stabilization of probiotic cells requires some specific processes with complex stages of food production and increased cost. The results of this study about the effectiveness of encapsulation to protect probiotics were controversial, and the high diversity of parameters under evaluation made it difficult to find the best method of encapsulation.

## CONFLICT OF INTEREST

The authors have declared no conflicts of interest for this article.

## AUTHOR CONTRIBUTIONS

**Zahra Motalebi Moghanjougi:** Data curation (equal); Formal analysis (equal); Investigation (equal); Resources (equal); Writing‐original draft (equal); Writing‐review & editing (equal). **Mahmoud Rezazadeh Bari:** Conceptualization (equal); Project administration (equal); Resources (equal); Supervision (equal). **Mohammad Alizadeh:** Conceptualization (equal); Investigation (equal); Software (equal); Supervision (equal); Validation (equal); Writing‐original draft (equal); Writing‐review & editing (equal). **Saber Amiri:** Conceptualization (equal); Investigation (equal); Methodology (equal); Software (equal); Writing‐original draft (equal); Writing‐review & editing (equal). **Hadi Almasi:** Conceptualization (equal).

## ETHICS APPROVAL

This study does not involve any human or animal testing.
